# Surface Charge Regulation of Graphene by Fluorine and Chlorine Co‐Doping for Constructing Ultra‐Stable and Large Energy Density Micro‐Supercapacitors

**DOI:** 10.1002/advs.202402033

**Published:** 2024-09-18

**Authors:** Binbin Liu, Jiagang Hou, Kai Wang, Caixia Xu, Qinghua Zhang, Lin Gu, Weijia Zhou, Qian Li, John Wang, Hong Liu

**Affiliations:** ^1^ Institute for Advanced Interdisciplinary Research (iAIR) Shandong Provincial Key Laboratory of Preparation and Measurement of Building Materials Collaborative Innovation Center of Technology and Equipment for Biological Diagnosis and Therapy in Universities of Shandong University of Jinan Jinan Shandong 250022 P. R. China; ^2^ Department of Materials Science and Engineering National University of Singapore Singapore 117574 Singapore; ^3^ Kyiv College at Qilu University of Technology Qilu University of Technology Shandong Academy of Sciences Jinan Shandong 250353 P. R. China; ^4^ Institute of Electrical Engineering Chinese Academy of Sciences Beijing 100190 P. R. China; ^5^ Institute of Physics Matter Physics Chinese Academy of Sciences/Beijing National Laboratory for Condensed Beijing 100190 P. R. China; ^6^ State Key Laboratory of Crystal Materials Shandong University Jinan Shandong 250100 P. R. China

**Keywords:** chlorine, co‐doped graphene, electrochemical exfoliation, flexible supercapacitors, fluorine

## Abstract

Settling the structure stacking of graphene (G) nanosheets to maintain the high dispersity has been an intense issue to facilitate their practical application in the microelectronics‐related devices. Herein, the co‐doping of the highest electronegative fluorine (F) and large atomic radius chlorine (Cl) into G via a one‐step electrochemical exfoliation protocol is engineered to actualize the ultralong cycling stability for flexible micro‐supercapacitors (MSCs). Density functional theoretical calculations unveiled that the F into G can form the “ionic” C─F bond to increase the repulsive force between nanosheets, and the introduction of Cl can enlarge the layer spacing of G as well as increase active sites by accumulating the charge on pore defects. The co‐doping of F and Cl generates the strong synergy to achieve high reversible capacitance and sturdy structure stability for G. The as‐constructed aqueous gel‐based MSC exhibited the superb cycling stability for 500,000 cycles with no capacitance loss and structure stacking. Furthermore, the ionic liquid gel‐based MSC demonstrated a high energy density of 113.9 mW h cm^−3^ under high voltage of up to 3.5 V. The current work enlightens deep insights into the design and scalable preparation of high‐performance co‐doped G electrode candidate in the field of flexible microelectronics.

## Graphical abstract

Co‐doping of the highest electronegative fluorine and large atomic radius chlorine into graphene (G) via a one‐step electrochemical exfoliation protocol, which can increase the repulsive force between nanosheets, enlarge the layer spacing of G, and increase active sites, is engineered to actualize the high capacitance and ultralong cycling stability with no structural pile for flexible micro‐supercapacitors.

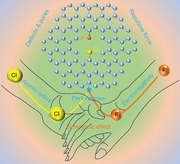

## 1 Introduction

Integratable high‐performance micro‐energy storage devices have attracted great focus in the field of various flexible wearable, portable, and biomedical microelectronics.^[^ ^1^ ^]^ In recent years, to actualize the high recyclability and large volumetric energy density, a plenty of efforts have been dedicated to exploiting high‐performance electrode material.^[^ ^2^ ^]^ Note that, graphene (G) and its derivatives as one class of the most popular two‐dimensional (2D) nanosheet material, have been demonstrated to be the ideal electrode materials to construct diversified micro‐supercapacitors (MSCs) owing to the high specific surface area, light weight, and striking electronic properties.^[^ ^3^ ^]^ Although great advances have been made to boost the performances of MSC through projecting diverse G, the 2D nanosheet itself easily undergoes the stacking and accumulation, which usually bring out the capacitance degradation during the long‐term utilization as well as the hard performance adjustment by G loading.^[^ ^4^ ^]^ Consequently, screening highly active and well‐dispersed G is very desirable to achieve ultra‐stable MSC with good reproducibility.

Electronegativity and atomic radius of the heteroatoms play an important role in modifying the electronic property of G, and thus influence the eventual capacitive performance.^[^ ^5^ ^]^ Compared with single‐element doping, co‐doping with two types of elements has been found to possess strong synergistic effect to improve the capacitive performances of G.^[^ ^6^ ^]^ Especially, fluorine (F) has the largest electronegativity, which may effectively attract the electron from the neighboring carbon (C) and form the polarized “ionic” C─F bond with rich negative charge on itself.^[^ ^7^ ^]^ Chlorine (Cl) not only has large electronegativity but also the larger atom radius. If achieving the co‐doping of F and Cl into G, the local charge accumulation initiated by the surface C─F and C─Cl bonds as well as the large Cl atom may effectively eliminate the G stacking through the repulsive role among nanosheets and possibly generate strong synergistic effects. It is inferred that the incorporation of halogen elements can more easily destroy the six‐membered ring structure to produce abundant defects and pores owing to the formation of the single bond with carbon and more effectively modify the charge storage properties for G. Liu et al. prepared co‐doped G with F and Cl heteroatoms through one‐step synthesis and used it in the field of oxygen reduction electrocatalysis.^[^ ^8a^ ^]^ The study showed that co‐doped G exhibited better stability than commercial Pt/C electrocatalysts and Cl‐doped G. The charge transfer resistance of co‐doped G is significantly lower than that of Cl‐doped G, indicating a possible synergistic interaction between F and Cl dopants. It is intriguing to achieve the controllable preparation of F/Cl co‐doped G nanosheets as well as explore their synergistic effect to dramatically improve the cycling stability of G as the electrode materials in MSCs.

At present, it is still a big challenge to prepare high‐quality G nanosheets simultaneously doped with two or more kinds of elements with a simple approach.^[^ ^8b^ ^]^ The traditional hydrothermal^[^ ^9^ ^]^ and chemical vapor deposition (CVD) methods^[^ ^10^ ^]^ to prepare mono‐ and multi‐elements doped G are not only time‐consuming but also require high temperature^[^ ^11^ ^]^ and multi‐step operation, commonly resulting in high production cost and unguaranteed quality. In this work, we proposed one simple, green, and scalable fabrication of high‐quality nanoporous F/Cl doped few‐layers G nanosheets through one step electrochemical exfoliation technique.^[^ ^12^ ^]^ During the electrochemical exfoliation process, simultaneous construction of rich pores as well as the F/Cl co‐doping with controllable doping ratios can be straightforwardly realized by only applying certain voltage over graphite flake in dilute sulfuric acid solution containing common F^−^‐ and Cl^−^‐ salt precursor. The porous structure alters the graphene's configuration, providing additional sites for energy storage.^[^ ^3c^ ^]^ Additionally, the electrochemical exfoliation method, as one form of defect engineering, also holds promising prospects for introducing defects in the manufacturing process of other materials. A facile template‐assisted method is employed to print interdigitated binder‐free electrode patterns on transparent polyethylene terephthalate (PET) substrate for assembly into flexible MSCs. Thereinto, co‐doped G suspensions are directly utilized to prepare metal/binder‐free interdigitated electrode patterns on PET substrates followed by constructing ultra‐thin and flexible MSCs with aqueous gel (polyvinyl alcohol/sulfuric acid (PVA/H_2_SO_4_)) and ionic liquid gel (1‐ethyl‐3‐methylimidazolium tetrafluoroborate with poly(vinylidene fluoride‐hexafluoropropylene) (EMIMBF_4_/PVDF‐HFP)), respectively. The as‐constructed aqueous gel‐based MSC with F/Cl co‐doped G as the electrode material achieves ultra‐long charge storage stability and versatile energy output by adjusting the integration mode. As demonstrated by density functional theory (DFT) calculations, the highest electronegative F into G endows the high surface electrovalence to increase the repulsive force between nanosheets in virtue of the formation of “ionic” C─F bond. Meanwhile, the introduction of large atomic radius Cl increases the active sites by accumulating the charge on the pore defects as well as enlarge the layer spacing of G. The strong synergy from the co‐doping of F and Cl overcomes the structure stacking of G nanosheets and enables its high charge storage performances. This work provides substantial insight into the synergistic effects between F and Cl as well as one effective avenue to strengthen the charge storage ability of G.

## 2 Results and Discussion

**Figure 1:**
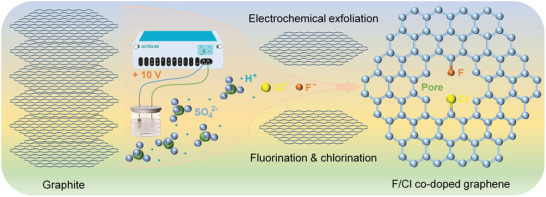
The preparation process of F/Cl co‐doped G.

**Figure** **1** illustrates the preparation process of few‐layers co‐doped G nanosheets. It is clear that merely economic graphite flake/rod is utilized as the source working electrode, while common mild sulfuric acid solution (0.1 m) containing ammonium fluoride and chloride was used as the electrolyte with an applied voltage of +10 V. The graphite electrode can be completely exfoliated into G nanosheets in a rapid rate within a few minutes. Compared with CVD^[^ ^13^ ^]^ and traditional liquid phase exfoliation methods,^[^ ^14^ ^]^ electrochemical exfoliation^[^ ^15^ ^]^ of bulk graphite possesses practical advantages in terms of simplicity and high efficiency without the use of high temperature, expensive instruments, and toxic organic reagents. In addition, the as‐made G possesses high dispersity into water, and the resultant suspension can be stored for a long period of more than one year with no agglomeration or sedimentation as shown in Figure S1 (Supporting Information), showing high solution stability and processability.

**Figure 2:**
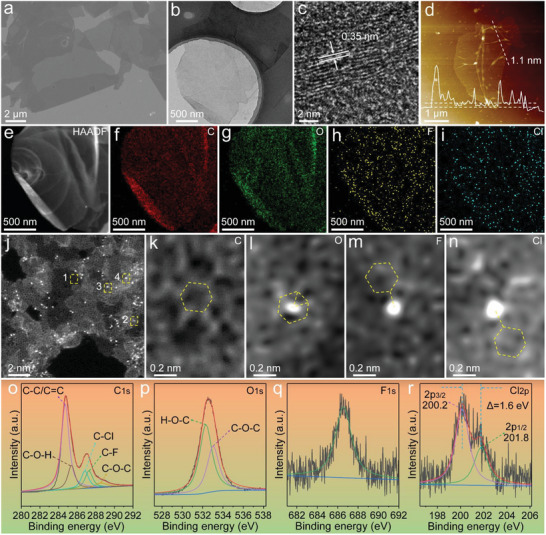
a) SEM, b) TEM, c) HRTEM, and d) AFM images of the F/Cl‐G nanosheets. e–i) TEM and EDS mapping images of the F/Cl‐G nanosheets. j) AC HAADF‐TEM image of the F/Cl‐G nanosheets. k–n) Focuses on the four different regions of 1–4 in Figure 2j. o) C 1s, p) O 1s, q) F 1s, and r) Cl 2p spectrum of the F/Cl‐G nanosheets.

The detailed morphology and structure of the resultant sample upon electrochemical exfoliation are shown in **Figure** **2**. The scanning electron microscopy (SEM) and low‐resolution transmission electron microscopy (TEM) images of the as‐prepared product in Figure 2a,b suggest the formation of 2D ultra‐thin and clear nanosheets with the large marginal size of up to ≈ 10 µm. Such feature can well inherit the intrinsic advantages of 2D G materials in terms of the high planar mass transport and mechanical flexibility. The high‐resolution (HR) TEM image in Figure 2c presents the ordered lattice fringes with the distance between the two fringes ≈ 0.35 nm. Figure 2d shows the atomic force microscopy (AFM) image with the contour curve of the ultrathin nanosheet, which represents the thickness of the as‐made nanosheets is only ≈ 1.1 nm, implying the thickness of two layers of C atoms.^[^ ^16^ ^]^ The energy dispersive spectroscopy (EDS) mapping images for one piece of the ultra‐thin nanosheet in Figure 2e–i testified the existence of four kinds of elements of C, oxygen (O), F, and Cl, which proves that the electrochemical exfoliation method can achieve the co‐doping of F and Cl elements into the G nanosheets. As shown in Figure S2 (Supporting Information), compared with those of pure exfoliated G (EG) without F or Cl, the diffraction peak of F and Cl co‐doped G (F/Cl‐G) shifts to a smaller angle of 25.81°, proving that the interlayer distance of co‐doped G enlarges upon introducing the F and Cl due to the much larger atomic radius of Cl.

In order to obtain more structural information of the few‐layer G‐based product, we performed aberration‐corrected high‐angle annular dark‐field transmission electron microscopy (AC HAADF‐TEM) characterization of the ultra‐thin nanosheets with atomic resolution as depicted in Figure 2j–n. From Figure 2j, it can be observed that some nanopores with the size around several nanometers distributed within the nanosheets, meanwhile there are a large number of concave‐convex areas on the surface, implying a certain degree of fluctuation in nanosheet thickness. Furthermore, a considerable number of bright spots can be discerned in the irregular six‐membered ring region, which are believed to be doped by the heteroatoms such as F and Cl. The irregularity in the six‐membered ring structure arises from the formation of single bonds between heteroatoms and C atoms, disrupting the original, more regular hexagonal configuration. As shown in Figure 2k, without the presence of bright spots, a part of C atoms presents a standard six‐membered ring structure, indicating that the bonding type of the C atom belongs to the planar sp^2^ hybridization. In addition, the existence of heteroatoms destroys this structure and forms the three‐dimensional (3D) sp^3^ hybridization, where this structural transformation from 2D planar to 3D solid will generate more structural defects on the surface of G derivatives.^[^ ^17^ ^]^ According to the different atomic radii and numbers of empty orbitals of O, F, and Cl atoms, it can be distinguished that the bright spots with weak brightness and small irregular spherical shape can be attributed to the O atoms along with the formation of C─O─C bonds (Figure 2l). Moreover, according to the radius of F and Cl atoms, it can be judged that the bright spot in Figure 2m is F, while the bright spot in Figure 2n is Cl. The introduction of halogen heteroatoms can effectively disrupt the original six‐membered ring structure of G, resulting in a large number of structural defects and nanopores, which will be beneficial for trapping the electrolyte ions and providing smooth multi‐dimensional channels for ion diffusion.

The surface chemical state of F/Cl‐G nanosheets was further investigated by X‐ray photoelectron spectroscopy (XPS). Figure S3 (Supporting Information),2o‐r revealed the presence of C, O, F, and Cl elements, indicating that F and Cl were successfully doped into G. The C1s core level region is shown in Figure 2o, the strong peak at 284.9 eV can be attributed to the C─C and C═C bonds in the graphite structure, while the peaks at 285.5, 287.2, 287.6, and 289.2 eV correspond to C─O─H, C─Cl, C─F, and C─O─C bonds. In the O1s spectrum in Figure 2p, the peaks at 532.7 and 533.4 eV correspond to C─O─H and C─O─C bonds, respectively. The energy spectrum of F1s in Figure 2q indicates the presence of F, where the binding energy at 686.7 eV implies the formation of C─F semi‐ionic bond.^[^ ^18^ ^]^ The orbitals of Cl 2p_3/2_ and 2p_1/2_ in Figure 2r show the peak positions at 200.2 and 201.8 eV with the difference ≈ 1.6 eV, signifying the formation of C─Cl covalent bonds.^[^ ^19^ ^]^ These observations further manifested that the F and Cl elements are indeed introduced into G nanosheet. In addition, the EDS results in Figure S4 (Supporting Information) also proved the presence of F and Cl elements in the samples. Based on the above characterization, it can be clearly found that in a 0.1 m dilute sulfuric acid solution containing F^‐^ and Cl^‐^ ions, this simple operation of electrochemical exfoliation of source graphite can effectively introduce F and Cl atoms into G.

In the process of graphite exfoliation, graphite will undergo structural expansion with the insertion of SO_4_^2−^, F^−^, Cl^−^, and OH^−^ ions, while newly formed gases enter into the expanded graphite layers under an applied voltage. Meanwhile, oxidation, fluorination, and chlorination processes occur at the same time, resulting in a certain number of heteroatoms attached to the dangling bonds at the edge of G.^[^ ^20^ ^]^ Moreover, the formation of these bonds can break the initial structure and generate rich nanopores, certain concave, and convex points, thereby increasing the specific surface area and the active sites of the few‐layer nanosheets, as well as the resultant energy storage properties.^[^ ^21^ ^]^

**Figure 3:**
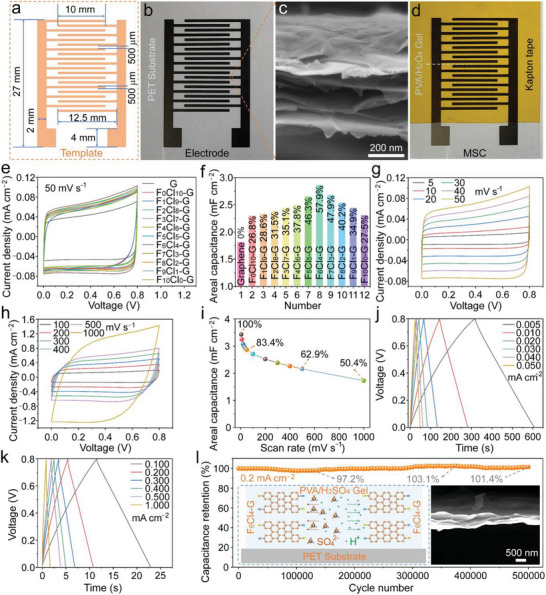
a) Schematic diagram of the metal template. b) Electrode on PET substrate. c) Cross‐sectional SEM image of electrode. d) G‐based flexible PVA/H_2_SO_4_‐MSC. Electrochemical test results of PVA/H_2_SO_4_‐MSCs. e) CV curves of MSCs constructed with different components of F/Cl‐G at the scan rate of 50 mV s^−1^. f) The areal specific capacitance of MSCs as a function of different components of F/Cl‐G at the scan rate of 50 mV s^−1^. g) CV curves of F_6_Cl_4_‐G‐MSC at the scan rate of 5–50 mV s^−1^. h) CV curves of F_6_Cl_4_‐G‐MSC at the scan rate of 100–1000 mV s^−1^. i) The areal specific capacitance of F_6_Cl_4_‐G‐MSC. j) GCD profiles of F_6_Cl_4_‐G‐MSC at the current density of 0.005–0.05 mA cm^−2^. k) GCD profiles of F_6_Cl_4_‐G‐MSC at the current density of 0.1–1 mA cm^−2^. l) Cycle stability of F_6_Cl_4_‐G‐MSC at the current density of 0.2 mA cm^−2^ for 500,000 uninterrupted charge‐discharge cycles (insert images: schematic diagram of electrolyte ion migration and SEM image of electrode after cycles).

In order to deeply explore the synergistic effect between F and Cl, the G nanosheets with different F and Cl ratios were made denoted as F_0_Cl_0_‐G, F_x_Cl_10‐x_‐G (x = 0, 1, 2, ···, 10). At present, the strategy of template‐assisted interdigitated electrode preparation has been established to be versatile and powerful for constructing planar micro‐devices, which can quickly complete the preparation of a single electrode and integrated circuits by adjusting the template. As shown in **Figures** **3**a and S5 (Supporting Information), a marked metal template with the corresponding dimensions were fabricated, where the distance between the two fingers is as small as 500 µm. A flexible electrode pattern shown in Figure 3b has the completely same size as the metal template through the template‐assisted transfer printing. The surface of the flexible electrode is relatively flat, and the G sheet structure can also be observed in Figure S6 (Supporting Information). As shown in Figure 3c, the cross‐section of the G electrode, which has a thickness of ≈ 277 nm, presents a layer‐by‐layer structure with the nanosheets stacked, in which the huge interspace can provide full contact between the G and the electrolyte. A flexible MSC can be constructed by coating the interdigitated regions of the G‐based electrode with PVA/H_2_SO_4_ aqueous gel (denoted as PVA/H_2_SO_4_‐MSC), which is presented in Figure 3d. Figure 3e shows the cyclic voltammetry (CV) curves of 12 flexible aqueous gel‐based MSCs constructed from different electrodes at the scan rate of 50 mV s^−1^. All CV curves present the roughly regular rectangle shape, indicating that the G material mainly possesses the electrical double‐layer capacitive (EDLC) behavior. Figure 3f shows the areal specific capacitance of each flexible device constructed with F_x_Cl_y_‐G in PVA/H_2_SO_4_ gel calculated according to the CV curve, in which all doped G samples perform better than undoped F_0_Cl_0_‐G‐MSC. All devices constructed by co‐doped G exhibit higher areal specific capacitance than mono‐element‐doped G. It is obvious that F_6_Cl_4_‐G‐MSC owns the best charge storage performances among these MSCs constructed by different co‐doped G materials, which is 57.9% higher than that of F_0_Cl_0_‐G‐MSC. From the capacitance change trend of these flexible MSCs, it is clear that the areal‐specific capacitance shows an upward trend with the increase of F content relative to Cl, and it shows a downward trend when it reaches the ratio of 6:4. This intriguing finding shows that any changes in the content of each element will affect the electronic conductivity (Table S1, Supporting Information) and the resulting performances of the as‐fabricated device. Only when the F/Cl ratio is the optimal, the MSC will exert the best charge storage performance, which substantially states that there are strong synergistic effects between co‐doped F and Cl. Therefore, F_6_Cl_4_‐G‐MSC with the highest areal‐specific capacitance is chosen as the typical role in subsequent research objects.

All the CV curves of F_6_Cl_4_‐G‐MSC in Figure 3g,h exhibit the typical EDLC behavior with the similar rectangular shape at the scan rates of 5–50 and 100–1000 mV s^−1^, respectively. The areal‐specific capacitance of the flexible MSC was calculated according to CV curves, and the relationship between the areal‐specific capacitance and scan rates is shown in Figure 3i. At a low scan rate of 5 mV s^−1^, the areal and volumetric specific capacitance of F_6_Cl_4_‐G‐MSC can reach the highest level of 3.46 mF cm^−2^ and 124.9 F cm^−3^, which also shows a superior rate performances with high capacitance retention of 83.4, 62.9, and 50.4% at the large scan rates of 50, 500, and 1000 mV s^−1^, respectively, implying a high mass transfer rate of this co‐doped ultra‐thin G nanosheets. Figure 3j,k show the galvanostatic charge‐discharge (GCD) profiles of F_6_Cl_4_‐G‐MSC at the current densities of 0.005–0.05 and 0.1–1.0 mA cm^−2^, respectively. It can be seen that all these profiles are nearly symmetrical triangle shapes, further testifying the EDLC behavior of the as‐made electrode. The relatively small voltage drops in the GCD profiles especially at high current densities suggest that the flexible MSC has excellent rate performance. As shown in Figure S7 (Supporting Information), the areal specific capacitance of F_6_Cl_4_‐G‐MSC was calculated to be 1.82 mF cm^−2^ at a current density of 0.005 mA cm^−2^ with a high capacitance retention of 83.8, 72.3, and 68.2% at the large current density of 0.05, 0.5, and 1.0 mA cm^−2^. The electrochemical impedance spectroscopy (EIS) data in Figure S8 (Supporting Information) exhibits the small equivalent series resistance (ESR) of 59.9 Ω in the high‐frequency region, further indicating the rapid mass transport of F_6_Cl_4_‐G. As we all know long‐term cycle stability is an important index to evaluate the practical application value of supercapacitors. As shown in Figure 3l, F_6_Cl_4_‐G‐MSC presents an ultra‐long cycling stability at the current density of 0.2 mA cm^−2^. Although the capacitance fluctuates between 97.2‐103.1% during cycling, it finally maintained at a high level of 101.4% relative to the original capacitance after as long as 500,000 cycles, indicating the stable structure characteristics of the F_6_Cl_4_‐G during the process of continuous ion adsorption and desorption. As shown in Figure S9 (Supporting Information),3l (insert image), the SEM image shows the cross‐sectional view of the electrode after 500,000 cycles, which presents no evident structure change compared with the electrode structure at the initial stage. It is regarded that such outstanding cycling stability benefits from the unique structure stability of the F/Cl‐G nanosheets. On one hand, the abundant pores and rugged surface allow the aqueous gel electrolyte to be fully filled, favoring for keeping the layered structure during repeated charge and discharge processes. Furthermore, the formation of C─F “ionic” bonds could generate strong repulsion in the nanosheet itself as well as between nanosheets. These specific structure features make F/Cl‐G nanosheets no structure staking and no collapse when building the electrode, thus achieving almost no performance attenuation during long‐term cycling.

**Figure 4:**
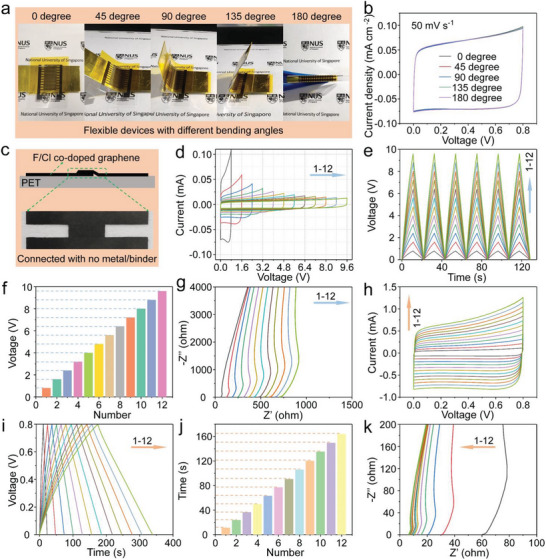
a) The flexible PVA/H_2_SO_4_‐MSCs with different bending angles. b) CV curves under different bending angles at the scan rate of 50 mV s^−1^. c) MSCs can connected with co‐doped G rather than conductive metal wire or binder. d) CV curves at the scan rate of 50 mV s^−1^ and e) GCD profiles at the current of 0.1 mA of 1–12 F_6_Cl_4_‐G‐MSCs in series. f) The relationship between the total voltage of an integrated circuit and the number of MSCs used for integration. g) The complex plane plot of 1–12 F_6_Cl_4_‐G‐MSCs in series. h) CV curves at the scan rate of 50 mV s^−1^ and i) GCD profiles at the current of 0.1 mA of 1–12 F_6_Cl_4_‐G‐MSCs in parallel. j) The relationship between the discharging time of an integrated circuit and the number of MSCs utilized for integration at the same current. k) The complex plane plot of 1–12 F_6_Cl_4_‐G‐MSCs in parallel.

The mechanical property of a flexible micro‐device is also an important evaluation parameter in regard to the requirements of various occasions. As shown in **Figure** **4**a, when bending the device into angles of 0 °, 45 °, 90 °, 135 °, and 180 °, the capacitance can be completely restored as high as that in a flat state with almost no obvious change after bending. Moreover, the CV curves and GCD profiles at different bending states almost overlap as shown in Figure 4b and Figure S10 (Supporting Information), indicating that the flexible device also has excellent mechanical flexibility. In integrated circuits, supercapacitors introduced often consume substantial space, prompting the need for efficient packaging to minimize their footprint in practical applications.^[^ ^3b^ ^]^ To tackle this challenge, we subjected the flexible device to roll‐up tests (Figure S11, Supporting Information). Results revealed that even after rolling, the device's capacitance remained nearly unaffected, providing further evidence of its outstanding flexibility and mechanical resilience (Figure S12, Supporting Information). Single micro‐device usually has the limitations in low voltage and small energy output, since it is necessary to design integrated circuits with various voltages and energy outputs to meet different requirements. A template‐assisted electrode preparation method was deployed to integrate multiple micro‐devices, which can avoid the use of any metal wires and binder and eliminate the lose certain performance of energy storage. The black tape‐shaped wire in a typical integrated circuit is shown in Figure 4c, in which the co‐doped G owns multiple functions in terms of storing the energy as an electrode material, connecting the different electrode patterns as a wire, and serving as a special binder to achieve a channel with low‐resistance for rapid migration of electrons because of the close fit between co‐doped G nanosheets. Consequently, such facile template‐assisted method can be used to prepare flexible interdigital electrodes with any series and parallel assembling on one piece of flexible PET substrate.

In order to explore the application of the flexible MSC in integrated circuits, 1–12 groups of flexible MSCs connected in series and parallel with different MSCs numbers were constructed. The connection of multiple devices in series can achieve the widening of the voltage window of the as‐made integrated circuit. It can be observed that the overall voltage becomes larger as the number increment of MSCs connected in series, indicating the well‐adjustable output for the MSCs. Figure 4d shows the nearly rectangular CV curves of the F_6_Cl_4_‐G‐MSCs from 1 to 12 devices in series. When the number of series devices reaches to 12, the overall voltage of the series circuit reaches a high level of 9.6 V. Figure 4e shows the GCD profiles with nearly symmetrical triangle shapes, for which the discharge time of these MSCs in series remains almost unchanged, indicating that the constructed interdigitated MSCs have a high degree of unity with the much stable energy output. The voltage of the integrated circuit will increase as the number of connections increases, that is, every time a device is added to the series circuit, the total voltage will increase by 0.8 V as shown in Figure 4f. This repeatability can also be reflected from the EIS results (Figure 4g), which shows an isometric manner with the increasing number of MSCs in series. Similar to the connection mode in series, high energy output can be obtained by connecting multiple devices in parallel. It can be seen from Figure 4h that with the number increases of the devices in parallel, the overall CV closed area increases in the form of an arithmetic sequence. As shown in Figure 4i,j, the GCD profiles at a current of 0.1 mA that the discharge time of 1–12 MSCs in parallel increases regularly as the number of parallel connections increases, which also manifests that the constructed MSCs have a high degree of unity. Figure 4k shows that the resistance will gradually become smaller as the number increases after being connected in parallel. All of the above electrochemical observations indicate that the as‐prepared flexible electrodes and the as‐constructed aqueous PVA/H_2_SO_4_‐MSCs have quite tiny individual differences, which are sufficient to meet the needs of integrated circuits, and present the potential application prospect in practical applications. In addition, in addition to using the template‐assisted transfer preparation method, a more precise laser etching method can also be used, that is, first preparing a complete co‐doped G thin layer, and then using a laser to directly etch it into the target micro‐integrated electrode pattern. Since the beam diameter of the laser can be set very small, the integrated circuit prepared by laser etching has higher precision and can achieve higher density integration. Moreover, because the integrated circuit made by laser has very few connection points, the device is not easily damaged when it is bent or rolled. Therefore, it is foreseeable that the co‐doped G thin layer can be integrated into highly foldable flexible devices after laser etching.

**Figure 5:**
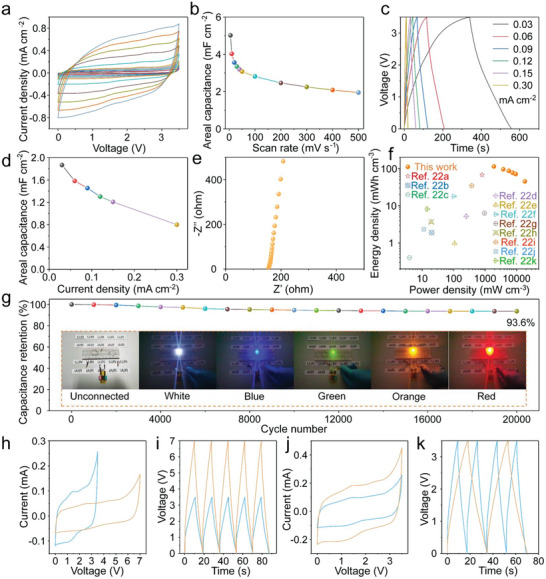
Electrochemical performance of EMIMBF_4_/PVDF‐HFP‐MSCs. a) CV curves at the scan rate of 5–500 mV s^−1^. b) The areal specific capacitance calculated from CV curves. c) GCD profiles at a current density of 0.03–0.3 mA cm^−2^. d) The areal specific capacitance calculated from GCD profiles. e) The complex plane plot of F_6_Cl_4_‐G based EMIMBF_4_/PVDF‐HFP‐MSC. f) Energy density of F_6_Cl_4_‐G based EMIMBF_4_/PVDF‐HFP‐MSC and other reported MSCs as a function of power density. g) Cycling stability of F_6_Cl_4_‐G based EMIMBF_4_/PVDF‐HFP‐MSC for 20,000 cycles at a current density of 0.3 mA cm^−2^ (Insets of Figure 5g). Demonstration of LED lighting by a single EMIMBF_4_/PVDF‐HFP‐MSC. h) CV curves and i) GCD profiles of the two F_6_Cl_4_‐G based EMIMBF_4_/PVDF‐HFP‐MSCs in series. j) CV curves and k) GCD profiles of the two F_6_Cl_4_‐G‐based EMIMBF_4_/PVDF‐HFP‐MSCs in parallel.

The aqueous gel‐based MSC exhibits a low energy density under the low voltage window (0–0.8 V), which are limited for the high voltage demand in field of various micro‐electronics. Therefore, in order to improve the working voltage of a single MSC, we select ionic liquid gel as the electrolyte to broaden the voltage window. The electrochemical tests of F_6_Cl_4_‐G‐MSC were carried out on the newly constructed flexible micro‐device with EMIMBF_4_/PVDF‐HFP ionic liquid gel (denoted as EMIMBF_4_/PVDF‐HFP‐MSC). As shown in **Figure** **5**a, the CV curves of the F_6_Cl_4_‐G‐based EMIMBF_4_/PVDF‐HFP‐MSC also shows a nearly rectangular shape at the scan rate of 5–500 mV s^−1^, implying its EDLC behavior in ionic liquid gel. As shown in Figure 5b, the F_6_Cl_4_‐G based EMIMBF_4_/PVDF‐HFP‐MSC exhibits a high areal and volumetric specific capacitance of 5.02 mF cm^−2^ and 181.2 F cm^−3^ at a scan rate of 5 mV s^−1^. When the scan rate is increased by 10 and 100 times to 50 and 500 mV s^−1^, its capacitance drops to 3.07 and 1.95 mF cm^−2^, which still remained 61.2 and 38.8% of capacitance compared with that at 5 mV s^−1^, indicating an excellent rate performance of this MSC. Compared with the ultrahigh rate performance of F_6_Cl_4_‐G based PVA/H_2_SO_4_‐MSC, the rate performance of ionic liquid‐based MSC became somewhat weakened because of the low transmission efficiency of ions in ionic liquid gel. Figure 5c shows the GCD profiles with nearly symmetrical triangle shapes of the F_6_Cl_4_‐G‐based EMIMBF_4_/PVDF‐HFP‐MSC at the current density of 0.03‐0.3 mA cm^−2^. However, as the charging process approaches a higher voltage of ≈ 3.5 V, the slope of the charging curve becomes smaller owing to the higher internal resistance in the device. Figure 5d shows the areal‐specific capacitance calculated from the GCD profiles. The highest areal and volumetric specific capacitance of the F_6_Cl_4_‐G based EMIMBF_4_/PVDF‐HFP‐MSC is 1.87 mF cm^−2^ and 67.5 F cm^−3^ at the current density of 0.03 mA cm^−2^ and 1.08 A cm^−3^, respectively. As the current density increases 10 times to 0.3 mA cm^−2^, the areal specific capacitance decreases to 0.80 mF cm^−2^ with 42.8% retained. The excellent rate performance of F_6_Cl_4_‐G‐based EMIMBF_4_/PVDF‐HFP‐MSC further indicates a relatively outstanding conductivity of the F_6_Cl_4_‐G ultra‐thin electrode. Figure 5e shows the ESR of the F_6_Cl_4_‐G based EMIMBF_4_/PVDF‐HFP‐MSC is only ≈ 154.1 Ω, signifying that the ions in the ionic liquid gel can migrate smoothly, thus enabling the MSC to obtain excellent performance in electrochemical energy storage. As shown in Figure 5f, our EMIMBF_4_/PVDF‐HFP‐MSC exhibits the maximum energy density of 113.9 mW h cm^−3^ at the power density of 1.9 W cm^−3^, and the maximum power density is 18.2 W cm^−3^ at the energy density of 45.2 mW h cm^−3^. In addition, we compared the performance of this MSC with recently reported G‐related MSCs and found that its performance surpasses those of the other reported micro‐devices.^[^ ^22^ ^]^ The long‐term cycling stability of the EMIMBF_4_/PVDF‐HFP‐MSC is displayed in Figure 5g. The areal‐specific capacitance retention rate is as high as 93.6% after 20,000 uninterrupted charge‐discharge cycles at a current density of 0.3 mA cm^−2^, indicating that the ultra‐thin F/Cl‐G can act as the electrode materials to construct very stable ionic liquid gel‐based MSC. Similar to aqueous gel, ionic liquid gel‐based devices also exhibit excellent mechanical property at different bending states as presented in Figures S13 and S14 (Supporting Information).

As presented above, the flexible EMIMBF_4_/PVDF‐HFP‐MSC possesses large areal‐specific capacitance, excellent rate performance, and long cycling stability. Due to the utilization of EMIMBF_4_/PVDF‐HFP ionic liquid gel to construct MSCs, the operating voltage of a single MSC has been raised to as high as 3.5 V. As shown in Figure 5g inset, one flexible MSC can easily light up a LED with different colors, such as white, blue, green, orange, and red, indicating the broad application prospects. Flexible EMIMBF_4_/PVDF‐HFP‐MSCs were also tested in series and parallel to boost the operating voltage and capacitance output. As shown in Figure 5h,i, the voltage of the circuit can be increased to 7.0 V by connecting two devices in series, showing great potential in practical applications with high voltage output. As shown in Figure 5j,k for the CV curves and GCD profiles of the MSCs connected in parallel, the voltage of the two MSCs connected in series and the discharge time of the two MSCs connected in parallel are twice of the single device, respectively, implying that the constructed MSCs are relatively uniform with no loss of capacitance during connections.

**Figure 6:**
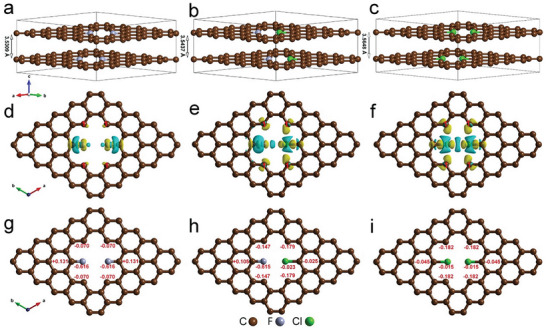
a–c) The optimized layer distances, d–f) charge density difference, and g‐i) Bader charge of F/F‐G, F/Cl‐G, and Cl/Cl‐G with the zigzag‐edge. C: brown, F: blue, Cl: green. The calculated charge density difference of F/F‐G, F/Cl‐G, and Cl/Cl‐G at isovalue = 0.005, which was calculated by the following equation: Δρ = ρ(total) – ρ(G) – ρ(Cl or F). The yellow region represents the electron accumulation, while the blue region represents the electrons depletion.

In order to further attest the origin of outstanding capacitance and stability of F/Cl‐G, DFT calculations were conducted to explore the synergistic effects for F and Cl co‐doping. As shown in **Figure** **6**a‐c, it is clear that the layer spacing of F/F‐G, F/Cl‐G, and Cl/Cl‐G gradually increased, which indicates that the atom radius of doping element will have some influence on the layer spacing.^[^ ^23^ ^]^ In addition, the charge density difference calculation shows that the C─F bonds in F/F‐G has the severe charge polarization due to the large electronegativity of F. It is evident that the electron accumulated on F atoms, and the linked C atoms has the electron depletion (Figure 6d), thus forming the so‐called “ionic” bond.^[^ ^24^ ^]^ It can be found that the formation of C─F bonds has little effects on the charge change of adjacent C atoms within the defect due to its small atom radius. From the Bader charge analysis, it is clear that F atom has the charge ≈ −0.616, while the neighboring C atoms within the defect have relatively lower charge (Figure 6g). As for the Cl/Cl‐G (Figure 6f), there is no evident electron polarization for C─Cl bonds despite its relatively large electronegativity, where their neighboring four C_1_ atoms as numbered 1 within the defect have the evident enrichment of electronics. It is considered that these four C atoms have two un‐bonded *p* orbitals with single electron, which can form the large delocalized *p‐p* π bond with adjacent Cl atoms owing to larger atom radius of Cl. From Figure 6i, it is clear that the four C_1_ atoms with broken bond possess the much larger Bader charge value. Consequently, it is inferred that the original electron pairs in Cl itself will interact with those of the adjacent C_1_ atoms to form the delocalized π bond with the electron accumulation by absorbing the electrons of ambient C atoms.^[^ ^23b^ ^]^ By comparison, the co‐doping of F and Cl combines their respective electron characteristics as shown in Figure 6e,h. It is obvious that in the defect region F atom has the evident charge polarization also along with the electron accumulation on upper and down adjacent C atoms, which is considered that the introduction of larger Cl atom facilitates the formation of delocalized π bond. The co‐doping of F and Cl can not only provide the repulsive forces between nanosheets but also produce rich highly active sites for charge storage. It can be concluded that the “ionic” C─F bond plays the important roles for reducing the sheet resistance, while the doping of Cl atom can facilitate the electron aggregation in the defect region upon the formation of the π cloud with p‐type doping to generate rich active sites as well as enlarge the layer spacing owing to its large atom radius.

We designed and successfully prepared high‐quality F/Cl co‐doped G nanosheets with the characteristics of large single nanosheet size, ultra‐thin thickness, abundant nanopores, and outstanding structure stability. On one hand, the strong electronegativity of F increases the repulsion of adjacent G nanosheets, and the large atom radius of Cl can increase the interlayer spacing of G. These structural features endow the F/Cl‐G suspension with ultra‐stable property with no sedimentation for more than one year. Furthermore, the as‐made flexible electrodes own the superb cycling stability without structure stacking. In addition, electrochemically exfoliating graphite and template‐assisted preparation of flexible electrodes is simple, eco‐friendly, and highly operable, which can furnish enlightened guidance for the screening of other G materials and the construction of flexible devices.

## 3 Conclusion

In summary, a facile and highly effective electrochemical exfoliation method was utilized to prepare high‐quality F and Cl co‐doped G nanosheets with few layers. During the rapid exfoliation of graphite, F^‐^ and Cl^‐^ ions in the solution simultaneously attack the dangling bond positions at the edge of G and around the nanopores, realizing the co‐doping of two kinds of halogen ions. Due to the porous structure and rich defects of co‐doped G, the as‐constructed MSCs based on F/Cl‐G shows high areal/volumetric specific capacitance, ultra‐long stability, and large energy density. Interdigitated electrodes with a pitch of only 500 µm by a template‐assisted method can be successfully to construct the flexible single MSC and integrated circuit with excellent electrochemical properties in both aqueous and ion liquid gel electrolytes.

## Conflict of Interest

The authors declare no conflict of interest.

## Supporting information

Supporting Information

## Data Availability

The data that support the findings of this study are available from the corresponding author upon reasonable request.
