# Supplementary material for: Surface Charge Regulation of Graphene by Fluorine and Chlorine Co‐Doping for Constructing Ultra‐Stable and Large Energy Density Micro‐Supercapacitors
Source: Adv Sci (Weinh). 2024 Sep 18;11(42):2402033. doi: 10.1002/advs.202402033 (PMC11558090; doi:10.1002/advs.202402033)
Supplement: Supplementary file 1 — Supporting Information [file ADVS-11-2402033-s001.pdf]

ADVANCED SCIENCE
Open Access

Supporting Information

for *Adv. Sci.*, DOI 10.1002/advs.202402033

Surface Charge Regulation of Graphene by Fluorine and Chlorine Co-Doping for Constructing Ultra-Stable and Large Energy Density Micro-Supercapacitors

Binbin Liu, Jiagang Hou, Kai Wang, Caixia Xu, Qinghua Zhang, Lin Gu*, Weijia Zhou, Qian Li, John Wang and Hong Liu**

Supporting Information

Surface charge regulation of graphene by fluorine and chlorine co-doping for constructing ultrastable and large energy density micro-supercapacitors

Binbin Liu[a,e], Jiagang Hou[b], Kai Wang[a,d], Caixia Xu[a], Qinghua Zhang[c], Lin Gu[c*], Weijia Zhou[a], Qian Li[a], John Wang[e], and Hong Liu[a, f*]*

[a]*Institute for Advanced Interdisciplinary Research (iAIR), Shandong Provincial Key Laboratory of Preparation and Measurement of Building Materials, Collaborative Innovation Center of Technology and Equipment for Biological Diagnosis and Therapy in Universities of Shandong, University of Jinan, Jinan 250022, Shandong Province, China*

[b]*Kyiv College at Qilu University of Technology, Qilu University of Technology, Shandong Academy of Sciences, Jinan 250353, Shandong Province, P. R. China*

[c]*Institute of Physics, Chinese Academy of Sciences/Beijing National Laboratory for Condensed, Matter Physics, Beijing, 100190 P. R. China*

[d]*Institute of Electrical Engineering, Chinese Academy of Sciences, Beijing, 100190 P. R. China*

[e]*Department of Materials Science and Engineering, National University of Singapore, 117574, Singapore*

[f]*State Key Laboratory of Crystal Materials, Shandong University, Jinan 250100, Shandong Province, China*

Fax: +86-531-82767033; Tel: +86-531-82767033

E-mail: chm_xucx@ujn.edu.cn; l.gu@iphy.ac.cn; ifc_liuh@ujn.edu.cn

Experimental Section

Electrochemical exfoliation of F/Cl-G nanosheets

The typical electrochemical exfoliation of graphite flake was conducted in a two-electrode system, in which a graphite flake is utilized as the working electrode with a platinum foil as the counter electrode in a mixed solution containing 0.1 M sulfuric acid, 0.1 M ammonium fluoride, and 0.01 M ammonium chloride. In the electrochemical exfoliation of graphite flake, a voltage of +10 V is applied under slight magnetic stirring for several minutes. The exfoliated F/Cl-G suspension was collected on a mixed cellulose ester (MCE) membrane (with a pore size of 0.22 μm) by filtration and washed several times with deionized (DI) water. The as-washed F/Cl-G sample was then dispersed in DI water (500 mL) by sonication for 1 h to obtain the suspension. The supernatant F/Cl-G suspension was slowly drawn out and put into another blue cap reagent bottle for subsequent characterization and flexible interdigitated electrode fabrication.

For the preparation process of co-doped graphene with different proportions of fluorine and chlorine, we control the F/Cl mixed solution with the total concentration of NH_4F and NH_4Cl as 0.01 M, with the ratios of F^- and Cl^- are 1:9, 2:8, 3:7, 4:6, 5:5, 6:4, 7:3, 8:2, and 9:1, respectively. In addition, a dilute acid solution containing only one kind of halogen and no halogen have also been prepared. For convenience, the solution containing only Cl^- is defined as 0:10, and the solution containing only F^- is defined as 10:0, and halogen-free solution is defined as 0:0. The samples obtained from the corresponding solutions were recorded as F_0Cl_0-G, F_0Cl_{10}-G, F_1Cl_9-G, F_2Cl_8-G, F_3Cl_7-G, F_4Cl_6-G, F_5Cl_5-G, F_6Cl_4-G, F_7Cl_3-G, F_8Cl_2-G, F_9Cl_1-G, and $F_{10}Cl_0$-G.

Characterization of F/Cl-G nanosheets

The morphology and structure of F/Cl-G nanosheets were characterized by field emission scanning electron microscopy (FESEM, MERLIN Compact, Carl Zeiss), high-resolution transmission electron microscopy (HRTEM, JEM-2100F), atomic force microscopy (AFM, Bruker Dimension Icon), powder X-ray diffractometer (XRD, Bruker D8 Advance, Cu-Kα, λ=0.15406 nm), X-ray photoelectron spectroscopy (XPS,

Kratos, AXIS Supra), and confocal microscopic Raman spectrometer (Raman, HORIBA LabRAM HR Evolution, λ=532 nm). The atomic structure of the F/Cl-G nanosheets was characterized using an ARM-200CF (JEOL, Tokyo, Japan) transmission electron microscopy operated at 200 kV and equipped with double spherical aberration (Cs) correctors. The electronic conductivity of the interdigitated electrode was determined using a four-point probe method.

Preparation of flexible interdigital electrodes

Regarding the preparation process of flexible interdigital electrodes, we continued to use the previous template-assisted transfer printing technology, and obtained electrode patterns with smaller interdigital spacing by replacing metal templates of different sizes. In short, the metal template (Figure S5) is covered on the water-based filter membrane, and a certain volume of co-doped graphene suspension is dropped. During reduced pressure filtration, water passes through the filter membrane and enters the filter bottle, while the graphene material remains in the hollow part of the template on the filter membrane to form an interdigitated electrode pattern. And then, we slowly removed the template, then covered the PET film on the filter membrane with the interdigitated electrode pattern, and squeezed out part of the water by applying a certain amount of pressure. Then the PET and filter membrane are separated, the graphene interdigitated electrode pattern is transferred to the PET film, and the preparation of the flexible electrode is completed.

Fabrication of all-solid-state MSCs

The PVA/H_2SO_4 hydrogel electrolyte was prepared by mixing 5 g of PVA and 5 g of sulfuric acid into 50 mL of deionized water, and heated at 80 ^oC for 2 h under stirring. For the preparation of ionogel electrolyte, 2 g PVDF-HFP polymer powder was fully dissolved in 10 mL acetone, and heated at 50 ^oC for 0.5 h under stirring. Then, 18 g ionic liquid of $EMIMBF_4$ was mixed with the solution of PVDF-HFP/acetone and stirred for 1 h at 50 ^oC. To fabricate MSCs, hot PVA/H_2SO_4 hydrogel electrolyte or $EMIMBF_4$/PVDF-HFP ionogel electrolyte was slowly drop-casted onto the projected area of the electrode patterns and solidified for 12 h.

The newly prepared graphene-based suspensions of various components were

used to prepare flexible electrodes on PET substrate, and PVA/H_2SO_4 (or EMIMBF_4/PVDF-HFP) was used as electrolyte to construct flexible micro-supercapacitors, which were denoted as F_0Cl_0-G-MSC, F_0Cl_{10}-G-MSC, F_1Cl_9-G-MSC, F_2Cl_8-G-MSC, F_3Cl_7-G-MSC, F_4Cl_6-G-MSC, F_5Cl_5-G-MSC, F_6Cl_4-G-MSC, F_7Cl_3-G-MSC, F_8Cl_2-G-MSC, F_9Cl_1-G-MSC, and $F_{10}Cl_0$-G-MSC. We can find out the optimal doping ratio of fluorine and chlorine by analyzing the performance of these devices under the same assembly method and electrochemical test conditions. The mass of each graphene-based electrode is controlled equally, and the mass of the electrode is adjusted by the volume of graphene ink.

Electrochemical measurements of MSCs

All electrochemical measurements were conducted over an electrochemical station of CHI 760E (Shanghai Chen Hua Instruments Co., China). Cyclic voltammetry (CV) and galvanostatic charge/discharge (GCD) were conducted in a potential window ranging from 0 to 0.8 V in the PVA/H_2SO_4 gel electrolyte, and in a potential range from 0 to 3.5 V in EMIMBF_4/PVDF-HFP gel electrolyte at different scan rates and current densities. The EIS was measured in the frequency ranging from 0.1 Hz to 100 kHz with AC amplitude of 5 mV.

Areal capacitance (C_{areal}, F cm^{-2}) and volumetric capacitance ($C_{volumetric}$, F cm^{-3}) of the MSCs were calculated by the CV curves according to equations (1) and (2):

$$C_{areal} = \frac{1}{v \cdot A \cdot (V_f - V_i)} \int_{V_i}^{V_f} I(V)dV \quad (1)$$

$$C_{volumetric} = \frac{1}{v \cdot V \cdot (V_f - V_i)} \int_{V_i}^{V_f} I(V)dV \quad (2)$$

Where v is the scan rate, A and V refer to the area and volume of electrode, V_f and V_i are the potential limits of CV curve, and $I(V)$ is the voltammetry discharge current, respectively.

In addition, the areal and volumetric capacitance can also be obtained from the GCD profile according to equations (3) and (4):

$$C_{areal} = \frac{I \cdot \Delta t}{A \cdot (V_f - V_i - IR)} \quad (3)$$

$$C_{volumetric} = \frac{I \cdot \Delta t}{V \cdot (V_f - V_i - IR)} \quad (4)$$

Where I is the discharge current, Δt refers to the discharge time, A and V refer to the area and volume of electrode, V_f and V_i are the potential limits of GCD profile, and IR is the Ohmic drop, respectively.

The volumetric energy density (E, Wh cm^{-3}) and power density (P, W cm^{-3}) of the MSCs were calculated from the equations (5) and (6):

$$E = \frac{1}{2} \times C_{volumetric} \times \frac{(V_f - V_i - IR)^2}{3600} \quad (5)$$

$$P = \frac{E}{\Delta t} \times 3600 \quad (6)$$

Where V_f and V_i are the potential limits of GCD profile, IR is the Ohmic drop, and Δt refers to the discharge time, respectively.

Calculation method

First-principle calculations were performed by the density functional theory (DFT) using the Vienna Ab-initio Simulation Package (VASP) package.[1] The generalized gradient approximation (GGA) with the Perdew-Burke-Ernzerhof (PBE) functional were used to describe the electronic exchange and correlation effects.[2] Uniform G-centered k-points meshes with a resolution of 2π*0.04 Å^{-1} and Methfessel-Paxton electronic smearing were adopted for the integration in the Brillouin zone for geometric optimization. The simulation was run with a cutoff energy of 500 eV throughout the computations. These settings ensure convergence of the total energies to within 1 meV per atom. Structure relaxation proceeded until all forces on atoms were less than 1 meV Å^{-1} and the total stress tensor was within 0.01 GPa of the target value. The DFT-D2 Van der Walls correction by Grimmie [3] was also considered in all calculations.

References

[1] G. Kresse, J. Furthmüller, *Comput. Mater. Sci.* **1996**, *6*, 15.

[2] a) J. P. Perdew, K. Burke, M. Ernzerhof, *Phy. Rev. Lett.* **1996**, *77*, 3865. b) P. E. Blöchl, *Phy. Rev. B* **1994**, *50*, 17953. c) G. Kresse, D. Joubert, *Phy. Rev. B* **1999**, *59*, 1758.

[3] a) S. Grimme, *J. Comput. Chem.* **2006**, *27*, 1787. b) S. Grimme, J. Antony, S. Ehrlich, H. Krieg, *J. Chem. Phys.* **2010**, *132*, 154104.

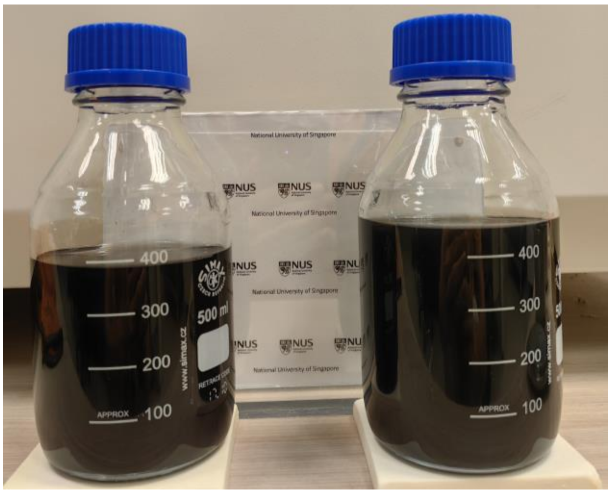

Figure S1 F/Cl co-doped graphene suspensions stored for over one year.

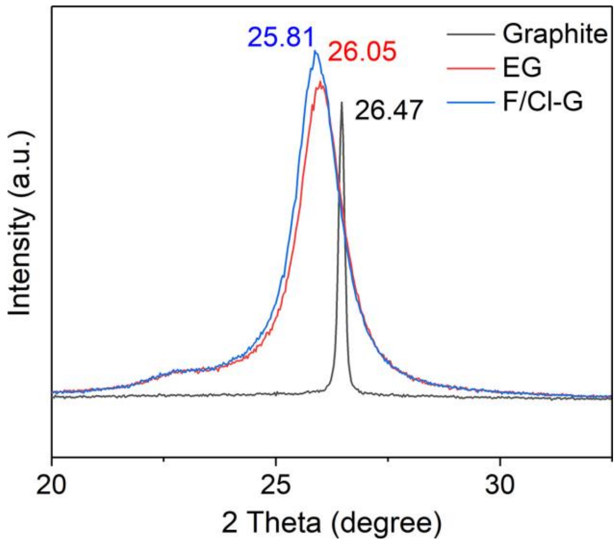

Figure S2 XRD pattern of the graphite, EG, and F/Cl-G nanosheets.

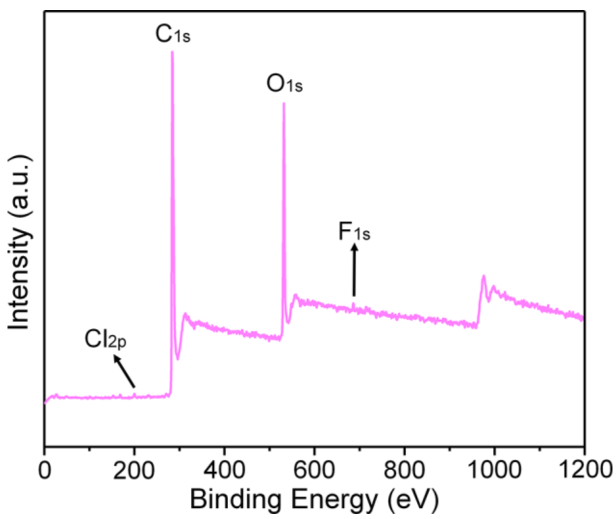

Figure S3 XPS data of the exfoliated F/Cl-G nanosheets.

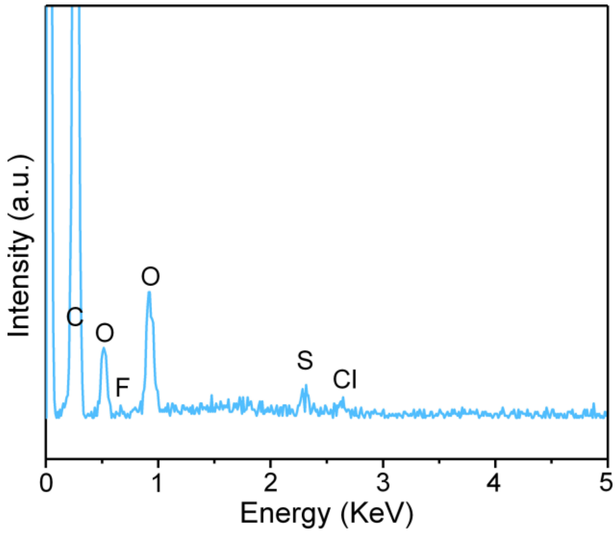

Figure S4 EDS of the F/Cl-G nanosheets.

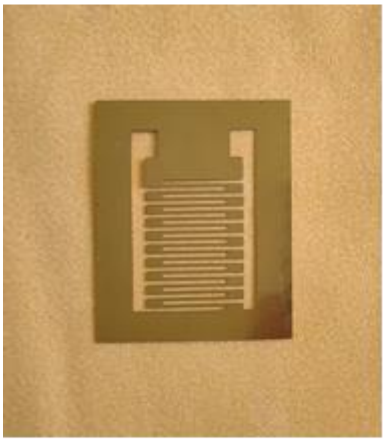

Figure S5 Optical photo of metal template.

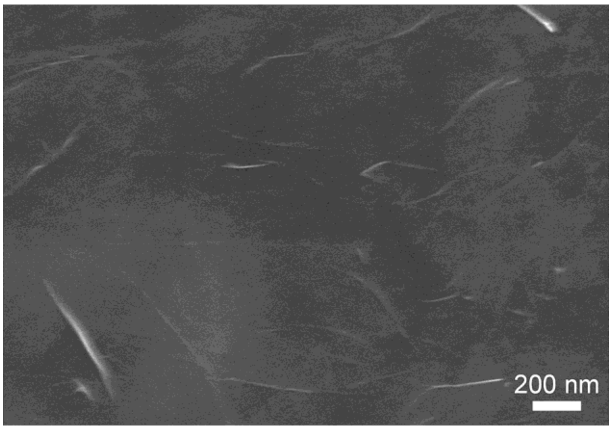

Figure S6 SEM image of the surface of the F/Cl-G electrode.

Table S1 The electronic conductivity of EG, Cl-G, F_2Cl_8-G, F_4Cl_6-G, F_6Cl_4-G, F_8Cl_2-G, and F-G flexible electrode.

Electrode	Average electronic conductivity (S cm^{-1})
EG	13.5
Cl-G	10.5
F_2Cl_8-G	10.7
F_4Cl_6-G	10.8
F_6Cl_4-G	10.9
F_8Cl_2-G	11.1
F-G	11.2

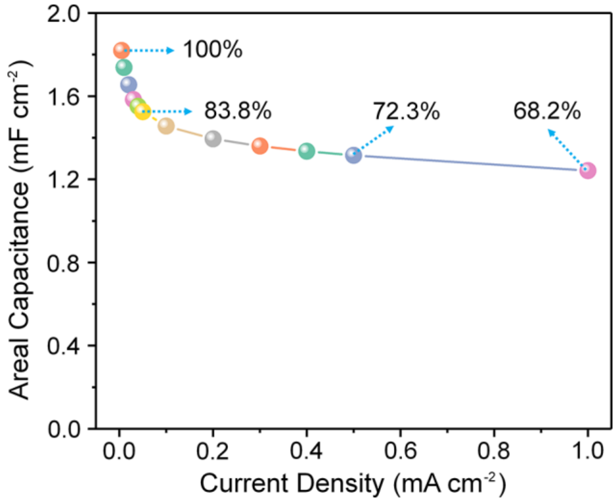

Figure S7 Areal capacitances of F_6Cl_4-G-MSC as a function of current density.

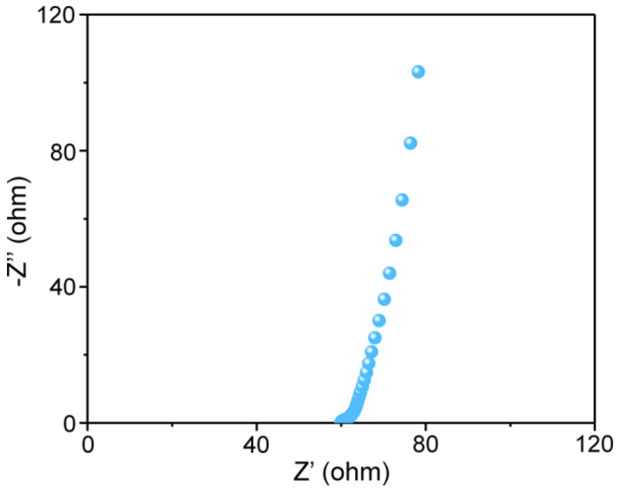

Figure S8 EIS of the F_6Cl_4-G based PVA/H_2SO_4-MSC.

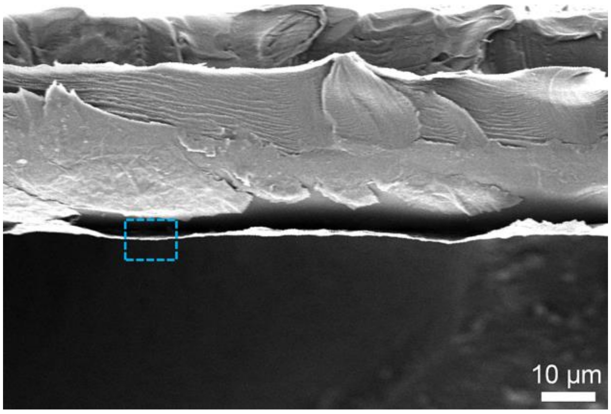

Figure S9 SEM image of the section of the F/Cl-G electrode on PET substrate.

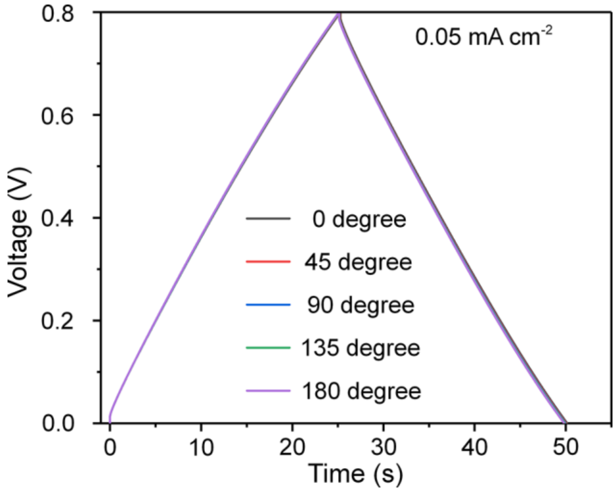

Figure S10 GCD profiles of F_6Cl_4-G based PVA/H_2SO_4-MSC obtained at the current density of 0.05 mA cm^{-2} under different bending angles.

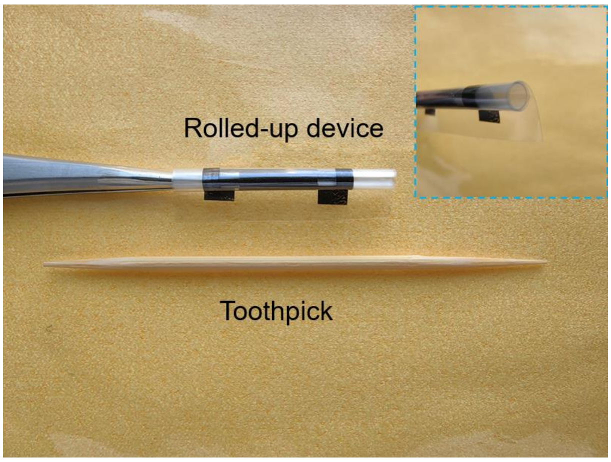

Figure S11 Optical photo of a rolled-up flexible MSC.

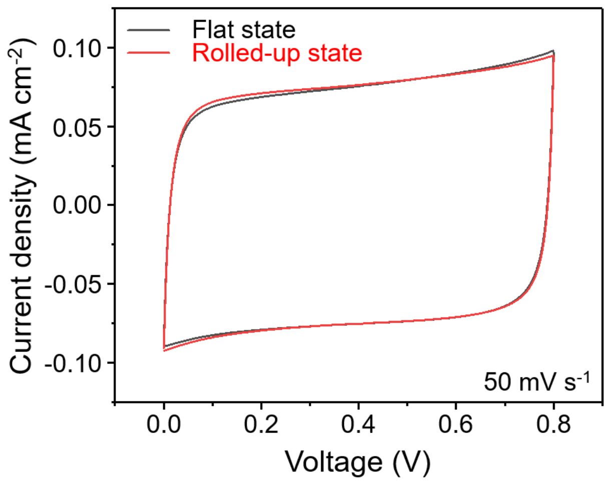

Figure S12 CV curves of F_6Cl_4-G-MSC in flat and rolled-up state at the scan rate of 50 mV s^{-1}.

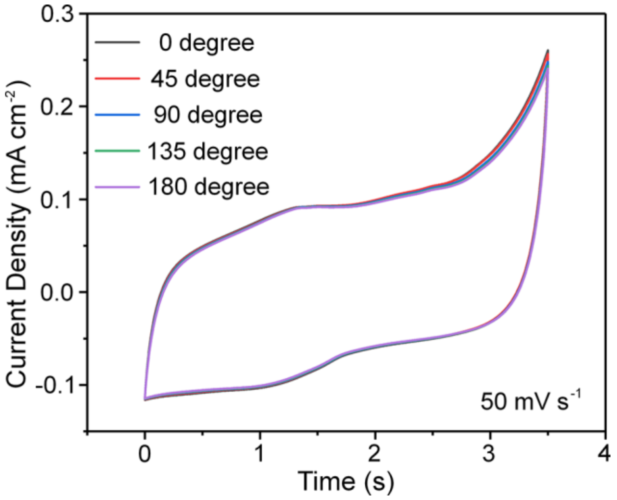

Figure S13 CV curves of F_6Cl_4-G based $EMIMBF_4$/PVDF-HFP-MSC obtained at the scan rate of 50 mV s^{-1} under different bending angles.

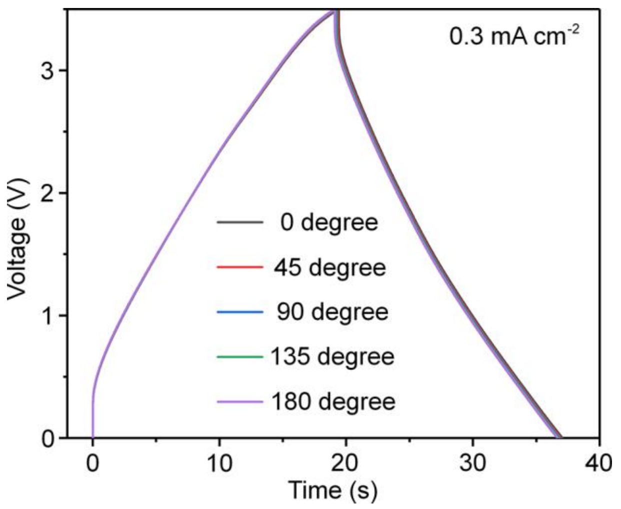

Figure S14 GCD profiles of F_6Cl_4-G based $EMIMBF_4$/PVDF-HFP-MSC obtained at the current density of 0.3 mA cm^{-2} under different bending angles.
